# Physiological exophoria did not increase the incidence of myopia in rural school children in Taiwan

**DOI:** 10.1097/MD.0000000000029482

**Published:** 2022-06-24

**Authors:** Jui-Hung Hsu, Li-Ju Lai, Tao-Hsin Tung, Wei-Hsiu Hsu

**Affiliations:** a School of Medicine, Kaohsiung Medical University, Kaohsiung, Taiwan; b Ophthalmology, Universal Eye Center, Chia-Yi, Taiwan; c School of Medicine, Chang Gung University, Tao-Yuan, Taiwan; d Department of Medical Research and Education, Cheng-Hsin General Hospital, Taipei, Taiwan; e Department of Orthopedics Surgery, Chang Gung Memorial Hospital, Chia-Yi, Taiwan.

**Keywords:** exophoria, myopia prevelance, new myopia

## Abstract

This study evaluated the incidence rate and risk factors for developing myopia in elementary school students in Chiayi, Taiwan. This prospective cohort study comprised 1816 students without myopia (grades 1 to 5 in Chiayi County). The students underwent a noncycloplegic ocular alignment examinations using an autorefractometer and completed a questionnaires at baseline and at a 1-year follow-up. A univariate logistic regression was used to assess the effects of the categorical variables on new cases of myopia. A multinomial logistic regression was then conducted. A chi-squared test was used to compare new cases of myopia in terms of ocular alignment. A Cox hazard ratio model was then used to validate factors associated with changes in ocular alignment. A *P* value of <.05 was considered significant. In 370 participants with new cases of myopia out of 1816 participants, a spherical error of −1.51 ± 0.6 diopters was noted at follow-up. The baseline ocular alignment was not a significant risk factor for developing myopia (exophoria vs orthophoria: OR 1.26, 95% CI 0.97–1.62; other vs. orthophoria: OR 1.15, 95% CI 0.73–1.82). However, new cases of myopia (HR 1.36, 95% CI 1.14–1.61), and baseline ocular alignment (exophoria vs orthophoria: HR 3.76, 95% CI 3.20–4.42; other vs orthophoria: HR 3.02, 95% CI 2.05–4.45) were associated with exophoria at follow-up. This study provided epidemiological data on the incidence of myopia in elementary school students in Chiayi, Taiwan. It also demonstrated that physiological exophoria does not predispose patients to developing myopia.

## 1 Introduction

The prevalence of myopia has increased rapidly over the past few decades and is expected to continuously rise, especially in East Asia.^[1–5]^ This increase offers an opportunity to investigate possible risk factors.^[6,7]^ Factors such as ethnicity, age, outdoor activities, the duration of near-work activity, and heterophoria have been associated with the prevalence of myopia.^[8–12]^

Heterophoria is an ocular deviation kept latent by the fusion mechanism. Near heterophoria can also result from a near binocular accommodative response.^[13]^ Binocular accommodative responses are most prevalent in participants with high exophoria at near vision.^[14]^ At approximately 50%, exophoria is highly prevalent at near fixation and is strongly associated with myopia in preschool and elementary students.^[12]^ Patients with pathologic myopia have a high prevalence of exophoria.^[15]^ Myopia has been suggested as a risk factor for concomitant exotropia.^[16]^ Conversely, intermittent exotropia has also been suggested as a risk factor for myopia.^[14]^ A population-based observational study demonstrated that children with intermittent exotropia exhibited a considerable tendency to develop myopia over time.^[17]^ Although the study established a close link between exophoria and myopia, little attention was given to whether physiological exophoria is a risk factor for myopia, or vice versa.^[14]^ Thus, this prospective cohort study analyzed the incidence of new cases of myopia and its risk factors, including exophoria and sleep duration, among students with myopia in a rural county in southern Taiwan. The study also analyzed the factors associated with exophoria 1 year later.

## 2 Methods

### 2.1 Participants

The school list was provided by Department of Education, Chia Yi County government. Chia Yi county is located at an suburban area in Taiwan. All students in the selected school were recruited for this investigation. The socio-economic scale was not provided by schools because of the vulnerability and sensitivity. This prospective cohort study was conducted across 26 elementary schools from September 2015 to January 2017 in Chiayi, Taiwan. The 5417 participants were aged 7 to 11 years and underwent examinations for refraction and eye health as well as interviews about lifestyle factors. The cohort included students without myopia. Refraction and eye health examinations were repeated the following year to determine the incidence of new cases of myopia and associated risk factors. Furthermore, ocular alignments were also measured the following year to reveal the factors associated with horizontal heterophoria.

### 2.2 Informed consent form

The local administration of the Education and School Board were contacted to request their cooperation. This study was approved by the Institutional Review Board of Chang Gung Foundation (Number: 201700887B0C501). It followed the tenets of Declaration of Helsinki. Informed consent was obtained from the parents or guardians of all children before the examination.

### 2.3 Eye examination

All students were screened in the same environment: a classroom with blackout curtains in the selected school. To reduce individual error, the same examiner team conducted all questionnaires and examinations. The questionnaires collected data on students’ gender, weight, height, sleep duration, eye-related symptoms, and history of exposure to atropine. Noncycloplegic refractive errors were assessed with an autorefractor (Autorefractometer ARK-1, Nidek Co., LTD., Aichi, Japan).^[6,18]^ Students with a history of ocular and physical pathologies, strabismus, or amblyopia were excluded. Refractive errors were defined by spherical equivalent (SE) refraction, calculated by adding the spherical diopter to one half of the cylindrical diopter. Myopia was defined as an SE of −1.0 diopter or greater in one or both eyes. Myopia was categorized as low myopia (−1.0 to −3.0 diopters) and high myopia (−3.0 diopters or less). Ocular alignment was assessed by observing corneal reflexes (Hirschberg test) and using the monocular cover-uncover test, in which each eye is briefly covered while the examiner watches for any movement in the opposite, uncovered eye that would indicate heterotropia. If no movement in the uncovered eye is noted, movement in the covered eye when the cover is applied and movement in the opposite direction (fusional movement) when the cover is removed indicates heterophoria. If the patient has heterophoria, the eye remains straight before and after the cover-uncover test; the deviation appears during the test as the result of an interruption of binocular vision. A participant with heterotropia, however, starts and ends the test with the same eye deviated, or in case of alternating heterotropia, ends the test with the opposite eye deviated.

This study included patients with SEs of less than −1.0 diopter. New myopia was defined as myopia of greater than −1.0 diopter. Patients were further analyzed regarding demographic data and risk factors with particular focus on ocular alignment. Adjusted odds ratios for all risk factors are presented.

### 2.4 Sample size

The sample size of each assessment outcome was calculated by using G∗power software version 3.1.9.7. (Heinrich Heine University, Dusseldorf, Germany). The input parameters used for the *z* test were alpha = 0.05, power= 0.8, P(Y = 1)= 0.2, and odds ratio= 1.18.^[19–21]^ The sample size was calculated as 1799.

### 2.5 Statistical analysis

The statistical analysis was conducted using SPSS Statistics 20.0. The data were presented as numbers (%) for fractions and as means with standard deviations for continuous variables where appropriate. A univariate logistic regression was used to assess the effects of the categorical variables on new cases of myopia. A multinomial logistic regression was then performed. In the categorical variable, the female, sleep time ≥8 hours/day, and orthophoria were the reference group. The division of new myopia cases in terms of ocular alignment was compared using the chi-squared test. A Cox hazard ratio model was then used to validate the associated factors for changes in ocular alignment. A *P* value of <.05 was considered significant.

## 3 Results

**Figure 1:**
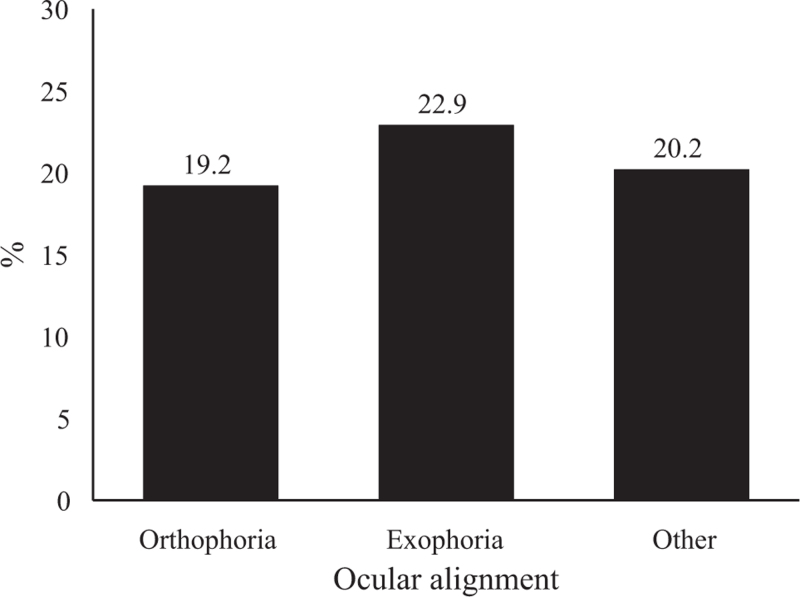
Total cohort, n = 1816; orthophoria, n  = 118; exophoria, n  = 667; others, n = 89; *P *= .213, chi-squared test.

A total of 5417 students aged 7 to 11 years were screened, including 1816 students without myopia. The data demonstrated an increase in height and BMI from baseline to the 1-year follow-up. Additionally, Table 1 reveals a difference (*P *< .05) in the distribution of different ocular alignments. For ocular alignment both at baseline and at follow-up, orthophoria was predominant, followed by exophoria and other ocular alignments. A total of 370 new cases of myopia in 1816 participants were identified with a spherical error of −1.51 ± 0.6 diopters at follow-up. Among them, 364 students exhibited low myopia, with a spherical error of −1.45 ± 0.44 diopters, and 8 students had moderate myopia, with a spherical error of −4.00 ± 1.07 (Table 2). The distribution of new myopia cases did not differ with regard to baseline ocular alignment (*P* > .05) (Fig. 1). The univariate analysis identified age (odds ratio [OR] 1.08, 95% CI 1.00–1.17), and height (OR 1.01, 95% CI 1.00–1.02), as factors associated with new cases of myopia (Table 3). The multinomial logistic regression confirmed that height (OR 1.01, 95% CI 1.00–1.02) was a risk factor for new myopia cases, whereas baseline exophoria was not a significant factor (Table 4).

**Table 1:** Demographic and clinical characteristics of participants (n = 1816).

	Baseline	Follow up	
Characteristic	Mean ± SD or n (%)	Mean ± SD or n (%)	*P*
Gender, male	967 (53.2)	967 (53.2)	
Age	8.88 ± 1.44	9.88 ± 1.44	
Body height (cm)	128.80 ± 17.60	136.91 ± 10.65	<.001
BMI (kg/m^2^)	18.23 ± 3.80	18.90 ± 4.15	<.001
Sleep, ≥8 h/day	1681 (92.6)	1690 (93.1)	.54
Ocular alignment			<.001
Orthophoria	1170 (64.4)	1080 (58.4)	
Exophoria	523 (29.3)	667 (36.7)	
Others	123 (6.8)	89 (4.9)	

**Table 2:** Demographic data of participants who developed myopia (n = 370).

	Mean ± SD or n (%)
Gender, (male)	185 (50.0)
Spherical equivalent (SE), OD (diopter)	−1.51 ± 0.60
Myopia	
Low myopia	362 (97.8)
Moderate myopia	8 (2.2)
Myopia Spherical equivalent (SE), OD (diopter)	
Low myopia	−1.45 ± 0.44
Moderate myopia	−4.09 ± 1.07

Definition of myopia: Low myopia SE, −1.00 diopters to −3.00 diopters, moderate myopia SE, −3.00 diopter to −6.00 diopter.

**Table 3:** Univariate analysis to compare the baseline characteristics of participants who developed myopia (n = 1816).

	Myopia vs No myopia	
	OR	95%CI of OR
Gender (male vs female)	0.85	0.68–1.07
Age	1.08	1.00–1.17
Body height (cm)	1.01	1.00–1.02
BMI (kg/m^2^)	1.00	0.97–1.03
Sleep (≥8 h/day vs <8 h/day)	1.03	0.66–1.56
Exophoria vs orthophoria	1.24	0.96–1.59
Other vs. orthophoria	1.13	0.71–1.78

The female, sleep time ≥8 h/day, and orthophoria were the reference group. CI = confidence intervals, OR = odds ratio.

**Table 4:** Multinomial logistic regression to compare the baseline characteristics of participants who developed myopia (n = 1816).

	Myopia vs No myopia	
	OR	95%CI of OR
Gender (male vs female)	0.87	0.69–1.10
Age	1.06	0.97–1.16
Body height (cm)	1.01	1.00–1.02
BMI (kg/m^2^)	0.98	0.95–1.02
Sleep (≥8 h/day vs <8 h/day)	1.00	0.64–1.55
Exophoria vs orthophoria	1.26	0.97–1.62
Other vs orthophoria	1.15	0.73–1.82

The female, sleep time≥8 h/day, and orthophoria were the reference group. CI = confidence intervals, OR = odds ratio.

Because the distribution of ocular alignment differed from baseline to follow-up, the factors associated with change in ocular alignment were analyzed using the Cox hazard model (Table 5). The results indicated that new myopia cases (HR 1.36, 95% CI 1.14–1.61), baseline ocular alignment (exophoria vs orthophoria, HR 3.76, 95% CI 3.20–4.42; other vs orthophoria, HR 3.02, 95% CI 2.05–4.45) were associated with follow-up exophoria.

**Table 5:** Cox proportional hazard model to compare the risk factors for ocular alignment in the following year (n = 1816).

	Exoophoria vs orthophoria	
	HR	95%CI of HR
Gender (female vs male)	0.86	0.74–1.00
Age	0.96	0.90–1.02
Body height (cm)	1.00	1.00–1.01
BMI (kg/m^2^)	1.00	0.98–1.03
Sleep (≥8 h/day vs <8 h/day)	0.93	0.69–1.24
Baseline ocular alignment		
Exophoria vs orthophoria	3.76^∗^	3.20–4.42
Other vs. orthophoria	3.02^∗^	2.05–4.45
New myopia (yes vs no)	1.36^∗^	1.14-1.61

Exophoria, n = 667, Orthophoria, n = 1060, Other, n = 89.

∗ *P* < .05.

## 4 Discussion

### 4.1 Incidence and risk factors for the development of myopia

This study revealed a rate of 20.4% per year for new cases of myopia in children aged 8 to 12 years in a rural area of southern Taiwan. This study corroborated recent research indicating a 20% to 30% incidence rate of myopia in an urban area in China.^[7]^ Furthermore, our study demonstrated that ocular alignment at baseline did not predispose students to developing myopia. In addition, new cases of myopia were associated with horizontal heterophoria both at follow-up and at baseline. The results revealed that horizontal heterophoria increased concomitantly with new cases of myopia. The temporal relationship between myopia and heterophoria is contested in the literature. Several studies have suggested that the increased convergence demand in children with heterophoria can result in myopia because of accommodative convergence.^[6,22–24]^ Other studies have indicated that the decreased accommodated load in people with myopia results in horizontal heterophoria because of accommodative convergence.^[25]^ Our study supports the second theory because our results indicated that horizontal heterophoria increased concomitantly with new cases of myopia, which is in contrast to the first theory.

This study identified a close link between horizontal heterophoria and myopia. Theoretically, both eyes assume a physiological position of rest in the absence of accommodative adaptation. The state of orthophoria may require a slight amount of accommodative convergence to maintain a parallel position of the eyes. In the absence of preexisting myopia, baseline ocular alignment was not associated with new cases of myopia. However, in children with myopia, the decreased accommodative demand could result in horizontal heterophoria because of accommodative convergence.^[6,22–24]^ This study demonstrated an increase in the incidence of exophoria along with new cases of myopia. The authors felt that this increased incidence could be related to different entities other than the physiological heterophoria shown in baseline ocular alignment. These differences may be important for identifying the role of heterophoria in the progression of myopia.^[26]^ However, we were unable to address this issue in this study. Clarifying the link between myopia progression and heterophoria requires further study.^[14]^

## 5 Methodological considerations

Although using a community-based cohort study design can clarify the temporal relationship between exophoria and other potential risk factors in the development of myopia, this study had some limitations. First, this study used noncycloplegic refraction. Cycloplegia is not well accepted by children and parents. Choong et al reported that autorefractors have a tendency toward overcorrection under noncycloplegic conditions.^[27]^ Therefore, in this study, myopia was defined as −1.0 diopter. Compared with the best-corrected visual acuity test and the cycloplegic refraction test, the noncycloplegic autorefraction test proved more convenient for visionscreening.^[7,18]^ Second, we assumed that all of the new cases of myopia occurred in January 2017. Because additional myopia cases could have occurred in subsequent years, the incidence of myopia may have been underestimated. Third, because of the short follow-up period the sample size was not sufficiently large to estimate the effects of potential prognostic factors on the progression of myopia. Additional long-term studies should be conducted to explore morbidity and biological plausibility. Finally, the study sample was from an Asian population. More studies are required to determine if exophoria increases the risk of myopia in Caucasian patients.

## 6 Conclusion

In conclusion, the study found a 20.3% incidence of new myopia in children aged 7 to 11 years in a rural area of Taiwan with an initial mean SE of −1.51 ± 0.6 diopters. Heterophoria did not increase the incidence of new myopia cases.

## Author contributions

LJL and JHH participated in the design of the study, collected data, performed the statistical analysis and drafted the manuscript. WHH participated in the design of the study and revision of the manuscript. WHH and THT participated in the study and statistical analysis. All authors read and approved the final manuscript.

**Conceptualization:** Jui-Hung Hsu, Li-Ju Lai, Wei-Hsiu Hsu.

**Data curation:** Jui-Hung Hsu, Wei-Hsiu Hsu.

**Investigation:** Li-Ju Lai.

**Methodology:** Li-Ju Lai, Tao-Hsin Tung.

**Writing – original draft:** Jui-Hung Hsu, Li-Ju Lai, Wei-Hsiu Hsu.

**Writing – review & editing:** Li-Ju Lai, Tao-Hsin Tung, Wei-Hsiu Hsu.
